# Supplementary material for: A DltE–DltD–DltX interaction network regulates lipoteichoic acid D-alanylation and Lactiplantibacillus plantarum-mediated growth promotion in Drosophila
Source: J Biol Chem. 2026 Sep 8;302(10):113522. doi: 10.1016/j.jbc.2026.113522 (PMC13653941; doi:10.1016/j.jbc.2026.113522)
Supplement: Supplementary file 1 [file mmc1.docx]

**SUPPORTING INFORMATION**

**A DltE–DltD–DltX interaction network regulates lipoteichoic acid D-alanylation and *Lactiplantibacillus plantarum*-mediated growth promotion in *Drosophila***

Renata C. Matos^1^, Nikos Nikolopoulos^2^, Quentin Perrier^1^, Xavier Robert^2^, Virginie Gueguen-Chaignon^3^, Hiroto Hirayama^4,5^, Jean-Pierre Simorre^6^, François Leulier^1^, Christophe Grangeasse^2^, Yann Guerardel^4,5^ and Stéphanie Ravaud^2,*^

^1^ Institut de Génomique Fonctionnelle de Lyon, École Normale Supérieure de Lyon, CNRS UMR 5242, Lyon 1 Université Claude Bernard, Lyon, France.

^2^ Molecular Microbiology and Structural Biochemistry, Lyon 1 Université Claude Bernard, CNRS UMR 5086, Lyon, France.

^3^ Protein Science Facility, CNRS UAR3444, INSERM US8, Université Claude Bernard Lyon 1, Ecole Normale Supérieure de Lyon, Lyon, France

^4^ Institute for Glyco-Core Research (iGCORE), Gifu University, Gifu, Japan

^5^ CNRS, IRL 2045, GlycoMIRAL, Nagoya University, Nagoya, Japan

^6^Institut de Biologie Structurale, Université Grenoble Alpes, CEA, CNRS UMR 5075, 3800, Grenoble, France

* Corresponding address: correspondence should be addressed to Stephanie Ravaud (stephanie.ravaud@univ-lyon1.fr)

**Supporting Tables**

**Table S1.** Data collection and refinement statistics.

|  | **DltD_extra_** |
| --- | --- |
| **Wavelength (Å)** | 0.98013 |
| **Resolution range (Å)** | 44.33 - 2.3 (2.382 – 2.3) |
| **Space group** | C 1 2 1 |
| **Unit cell (Å, °)** | 147.61, 85.33, 110.83, 90.00, 90.01, 90.00 |
| **Total reflections** | 438603 (23783) |
| **Unique reflections** | 61272 (6081) |
| **Multiplicity** | 6.9 (5.7) |
| **Completeness (%)** | 99.93 (99.98) |
| **Mean I/sigma(I)** | 10.4 (1.1) |
| **Wilson B-factor** | 42.61 |
| **R-merge** | 0.193 (1.590) |
| **R-meas** | 0.208 (1.746) |
| **R-pim** | 0.078 (0.707) |
| **CC1/2** | 0.995 (0.442) |
| **Reflections used in refinement** | 61272 (2780) |
| **Reflections used for R-free** | 3063 (139) |
| **R-work** | 0.1788 (0.3287) |
| **R-free** | 0.2166 (0.3294) |
| **Number of non-hydrogen atoms** | 9701 |
| Macromolecules | 9429 |
| Ligands | 18 |
| Solvent | 254 |
| **Protein residues** | 1166 |
| **RMS(bonds)** | 0.003 |
| **RMS(angles)** | 0.568 |
| **Ramachandran favored (%)** | 96.47 |
| **Ramachandran allowed (%)** | 3.45 |
| **Ramachandran outliers (%)** | 0.09 |
| **Rotamer outliers (%)** | 1.49 |
| **Clashscore** | 3.45 |
| **Average B-factor** | 49.28 |
| Macromolecules | 49.31 |
| Solvent | 47.78 |
| Ligand | 58.87 |

Statistics for the highest-resolution shell (2.382-2.3 for data collection and 2.34-2.3 for refinement statistics) are reported in parentheses.

**Table S2.** Bacterial strains and plasmids used in this study.

| Strain or plasmid | Relevant characteristics | Reference or source |
| --- | --- | --- |
| Strain |  |  |
| *E. coli* |  |  |
| TG1 | supE hsd5h thi (∆lac-proAB) F’ (traD36 proAB-lacZ∆M15) | (36) |
| GM1674 | dam-dcm- repA+ | (37) |
| *L. plantarum* |  |  |
| NC8 | Isolated from grass silage, plasmid free | (38) |
| *∆dltD* | NC8 strain deleted for nc8_1733 | This study |
| *dltD-6M* | Knock-in of *dltD-6M*X sequence on ∆*dltD* mutant | This study |
| *∆dltX* | NC8 strain deleted for nc8_1737 | This study |
| *dltX_∆cterm_* | NC8 strain deleted for *dltX* c-terminal sequence | This study |
| Plasmids |  |  |
| pG+host9 | Ermr, repATs | (39) |

**Table S3. Primers used in this study.**

| Primer name | Sequence (5’→3’)* | Reference |
| --- | --- | --- |
| OL01 | AGTGGATCCCCCGGGCTGCAGTGTGATGTACGTCTATAGTATGGA | This study |
| OL02 | TAGGTTATTCGTTCATTGCAAGTTCTCAACCT | This study |
| OL03 | TGCAATGAACGAATAACCTACTAAAAAGTACAGGCCA | This study |
| OL04 | CTTGATATCGAATTCCTGCACCTTCATGAATCCGAACACTCA | This study |
| OL05 | AAAGTATCTCGGGTTAGTTG | This study |
| OL06 | ATGCTAGCCGTAATGTAACC | This study |
| OL07 | CTTGATATCGAATTCCTGCACGTCAAAGTTGGCAAGTTTTCA | This study |
| OL08 | ATGGCATGTTTTTTTAAAAATGGATGCAGTAACACT | This study |
| OL09 | CATTTTTAAAAAAACATGCCATCACCTCCTCC | This study |
| OL10 | AGTGGATCCCCCGGGCTGCACGATTCAAAAGTCAATGACGGC | This study |
| OL11 | AACGCTTCGCCAAATGTTAG | This study |
| OL12 | CCCGTACGTATTGAAGATCC | This study |
| OL13 | TGGATCCCCCGGGCTGCACTTTGGTACGCGGATGCTAC | This study |
| OL14 | ATATGGTTAGATCCAAATGAGAACGGTGAAAGC | This study |
| OL15 | TCATTTGGATCTAACCATATTTCTAAGGATACTGAGA | This study |
| OL16 | TTAGTAGGTTATTCAAAATCCTTGATTTCACTGGGT | This study |
| OL17 | AGGATTTTGAATAACCTACTAAAAAGTACAGGCCA | This study |
| OL18 | TGATATCGAATTCCTGCAATAATCCCGTGCTAACGGTT | This study |
| OL19 | GCGGTTCGTTAAGGATTACG | This study |
| OL20 | TATCACCTACTCGGGTATCC | This study |
| OL21 | TGGATCCCCCGGGCTGCACAACGCTTCGCCAAATGTTAGA | This study |
| OL22 | CCATTTTTACTTCGCTTGGTTGATCCCAC | This study |
| OL23 | CAAGCGAAGTAAAAATGGATGCAGTAACACT | This study |
| OL24 | TGATATCGAATTCCTGCACGTCAAAGTTGGCAAGTTTTCA | This study |
| OL25 | GGTTGGCATTATGCAGTTGG | This study |
| OL26 | CAATTTGAGTTACCGCGACG | This study |

 *Overlapping sequences for Gibson assembly are underlined.

**Supporting Figures**

**Figure S1.** **Production of DltD_extra_ and DltE_extra_.** **A,** DltD is composed of a transmembrane segment predicted between residues 7 and 30 and a larger C-terminal extracellular region from residues 30 to 425 and named DltD_extra_. **B,** SDS PAGE analysis of the purity of DltD_extra_ after a two-step purification procedure including an ion exchange chromatography (IEX) and a size exclusion chromatography. The band corresponding to DltD_extra_ is shown by an arrow. **C,** Size exclusion chromatography (SEC) and elution profile after injection of the IEX-purified DltD_extra_ on a Superdex 200 10/300 GL. The blue line corresponds to the SEC fractions labeled on the SDS-PAGE. **D,** DltE is composed of a transmembrane segment predicted between residues 12 and 34 and a larger C-terminal extracellular region from residues 34 to 397 and named DltE_extra_. **E**, SDS PAGE analysis of the purity of DltE_extra_ after a two-step purification procedure including a Ni-Affinity and a size exclusion chromatography (13). The band corresponding to DltE_extra_ is shown by an arrow. **F,** Size exclusion chromatography (SEC) and elution profile after injection of the Ni-affinity purified DltE_extra_ on a Superdex 200 10/300 GL. The blue line corresponds to the SEC fractions labeled on the SDS-PAGE.

**Figure S2.** **DltD sequence conservation.** Sequence alignments between *L. plantarum* DltD and DltD from *Ef, Enterococcus faecalis; Sp, Streptococcus pneumoniae; Ll, Lactococcus lactis; Bs,Bacillus subtilis; Lm,Leuconostoc mesenteroides; Sa, Staphylococcus aureus*. The secondary structure extracted from the 3D X-ray structure of DltD_extra_ (this study) is depicted above. The three catalytic residues are marked with a red star while the 3 other conserved residues of the active site are marked with a yellow star. The DltX interacting residues are marked with orange triangles and lines.

**Figure S3. LTA Fragments docked in DltD_extra_ structure. A,** Docking poses of LTA fragments containing three (in pink) or four (in green) glycerol-phosphate repeats with favorable docking scores mapped onto the crystal structure of DltD_extra_. The protein is shown as a cartoon representation, while the docked ligands are displayed as surfaces. The orientation is the same as in Figure 2, and conserved active-site residues are colored as in Figure 2. The ensemble of docking poses suggests a possible trajectory along the DltD surface that could accommodate the processing of longer LTA chains. **B,** 3D representations of the triglycerol phosphate and tetraglycerol phosphate molecules used in the docking experiments to mimic LTA fragments. **C,** Surface representation of DltD_extra_ in the same orientation as in A, colored according to electrostatic potential. **D,** The predicted binding poses of LTA fragments (as in A) preferentially associate with positively charged regions of the DltD protein surface (colored as in C).

**Figure S4. The DltD-DltX structural model. A,** AF3 model of the DltD-DltX complex and the corresponding PAE matrix. The predicted local Distance Difference Test (plDDT) is a metric of model confidence, with plDDT values > 90 representing very high confidence, 70–90 representing moderate confidence, 50–70 representing low confidence, and < 50 representing very low confidence. **B,** The AlphaBridge plot for this predicted complex. **C,** The two interfaces are mapped on the structure of the complex in blue and orange.

**Figure S5.** ***Lp*DltX sequence and AF3 predicted structure.** *Lp*DltX is composed of a transmembrane segment predicted between residues 12 and 35 and a C-terminal extracellular extension of 14 residues. The sequence alignment between *L. plantarum* DltX and DltX from *Ef, Enterococcus faecalis; Sp, Streptococcus pneumoniae; Ll, Lactococcus lactis; Bs, Bacillus subtilis; Lm, Leuconostoc mesenteroides; Sa, Staphylococcus aureus* highlights the sequence conservation of the last 6 residues ^44^FIYNEF^49^. The orange line marks the region predicted to interact with DltD.

**Figure S6.** **The DltD_6MX_ mutant.** **A,** SDS PAGE analysis of the purity of DltD_6MX_ after a two-step purification procedure including Ni-Affinity and a size exclusion chromatography. The band corresponding to DltD_6MX_ is shown by an arrow. **B,** Size exclusion chromatography (SEC) and elution profile after injection after injection of the Ni-affinity purified DltE_extra_ on a Superdex 200 10/300 GL. The blue line corresponds to the SEC fractions labeled on the SDS-PAGE.**C.** MST normalized dose–response curves for the binding interaction between the C-terminal peptide of DltX and DltD_6MX_ mutant obtained by plotting ∆Fnorm against the concentration of the C-terminal peptide of DltX.

**Figure S7.** ^1^H,^15^N BEST-TROSY spectrum of the ^15^N-labeled DltE_extra_ protein.

**Figure S8. The DltE interaction with DltD and DltX.**

**A,** The DltE-DltX interaction was assessed by bacterial two-hybrid assay. A blue coloration indicates a positive interaction as depicted by the positive control (+) and the negative control (-) and the comparison with color obtained for the interaction between DltA and DltC that were previously shown to form a complex (14). **B,** MST normalized dose–response curve for the binding interaction between DltE_extra_ and the C-terminus of DltX were obtained by plotting ∆Fnorm against the concentration of the C-terminus of DltX. Plotting of the change in thermophoresis and concomitant fitting of the data yielded a *K_d_* in micromolar range. **C,** AF3 model of the DltE_extra_ interaction with the C-terminus of DltX (^44^FIYNEF^49^) and the corresponding PAE matrix. The predicted local Distance Difference Test (plDDT) is a metric of model confidence, with plDDT values > 90 representing very high confidence, 70–90 representing moderate confidence, 50–70 representing low confidence, and < 50 representing very low confidence.

**References**

36. Baer, R., Bankier, A.T., Biggin, M.D., Deininger, P.L., Farrell, P.J., Gibson, T.J., Hatfull, G., Hudson, G.S., Satchwell, S.C., Séguin, C., et al. (1984) DNA sequence and expression of the B95-8 Epstein-Barr virus genome. *Nature*. **310**, 207-11.

37. Palmer, B. R. and Marinus, M. G. (1994) The dam and dcm strains of *Escherichia coli*: a review. *Gene* **143**, 1–12.

38. Axelsson, L., Rud, I., Naterstad, K., Blom, H., Renckens, B., Boekhorst, J., Kleerebezem, M., van Hijum, S., and Siezen, R.J. (2012) Genome sequence of the naturally plasmid-free *Lactobacillus plantarum* strain NC8 (CCUG 61730). *J Bacteriol*. **194**, 2391-2

39. Maguin, E., Prévost, H., Ehrlich, S. D. and Gruss, A. (1996) Efficient insertional mutagenesis in *lactococci* and other gram-positive bacteria. *J Bacteriol* **178**, 931–935.
